# Potential markers for sample size estimations in hereditary spastic paraplegia type 5

**DOI:** 10.1186/s13023-021-02014-w

**Published:** 2021-09-19

**Authors:** Qianqian Lin, Ying Liu, Zhixian Ye, Jianping Hu, Wenjie Cai, Qiang Weng, Wan-Jin Chen, Ning Wang, Dairong Cao, Yi Lin, Ying Fu

**Affiliations:** 1 Department of Neurology and Institute of Neurology of First Affiliated Hospital, Institute of Neuroscience, Fujian Medical University, Fuzhou, 350005 China; 2 Fujian Key Laboratory of Molecular Neurology, Fujian Medical University, Fuzhou, 350005 China; 3 Department of Radiology of First Affiliated Hospital, Fujian Medical University, Fuzhou, 350005 China

**Keywords:** Spastic paraplegia, Hereditary, Magnetic resonance imaging, Biomarkers, Sample size

## Abstract

**Background:**

Aim to identify potential biomarkers to assess therapeutic efficacy for hereditary spastic paraplegias type 5 (SPG5) by investigating the clinical, cerebrospinal fluid (CSF) and magnetic resonance imaging (MRI) features.

**Methods:**

We performed a cross-sectional study to compare SPG5 patients with age- and sex-matched healthy controls who underwent conventional and quantitative MRI techniques of spinal cord (C1-T9) and brain. SPG5 patients also underwent assessment for clinical status and CSF biomarkers (27-hydroxycholesterol, neurofilament light). We identified a set of markers with standardized effect sizes (|t|> 0.5) to estimate sample sizes for disease progression (disease duration > 14 years vs. ≤ 14 years).

**Results:**

Seventeen genetically confirmed SPG5 patients (11 men, 6 women; age range, 13–49 years; median disease duration, 14 years) were enrolled. Compared to healthy controls, the total spinal cord area (SCA) of SPG5 patients was reduced particularly at the thoracic levels (cervical levels: 12–27%; thoracic levels 41–60%). Patients did not show significant alterations of brain signal abnormalities or atrophy relative to controls. A total of 10 surrogate markers were selected and a minimum sample size was achieved with the measurement of SCA on T9 (n = 22) much less that what would be required if using clinical disability assessment (n = 124).

**Conclusions:**

SPG5 patients showed distinct MRI features of spinal cord atrophy without significant brain alterations. Our finding supports the measurements of spinal cord on T9 level as potential endpoint for SPG5 clinical trials.

*Trial registration* ClinicalTrials.gov, NCT04006418. Registered 05 July 2019, https://clinicaltrials.gov/ct2/show/NCT04006418?term=NCT04006418&draw=2&rank=1.

**Supplementary Information:**

The online version contains supplementary material available at 10.1186/s13023-021-02014-w.

## Background

Hereditary spastic paraplegias (HSP) are a large, genetically diverse group of inherited neurologic disorders characterized by a length-dependent distal axonopathy of the corticospinal tracts, resulting in lower limb spasticity and weakness [1]. Hereditary spastic paraplegia type 5 (SPG5), a subtype of HSP, is caused by autosomal recessive loss-of-function mutations in *CYP7B1*, a gene that encodes oxysterol-7a-hydroxylase. This mutations leads to the accumulation of *CYP7B1* substrates, like 27-hydroxycholesterol (27-OHC), in plasma and cerebrospinal fluid (CSF) [2, 3].

A better understanding of SPG5 pathophysiological mechanisms could yield a mechanistic-approach therapy to modulate cholesterol metabolism in patients with SPG5. To date, two clinical trials explored hypercholesterolemia therapies (atorvastatin) in SPG5 patients with changes of 27-OHC in serum as primary endpoints [3, 4]. However, there is no definite evidence for a beneficial impact of 27-OHC serum levels reduction in SPG5 patients. Furthermore, the rarity of the disease, and the slow, and variable rates of disease progression hurdles development and assessment of efficacious therapies [3, 4]. Therefore, it is essential to identify sensitive and reliable biomarkers for disease progression in small-scale studies.

Recently, quantitative magnetic resonance imaging (MRI) and neurofilament light chain (NFL) emerged as valuable tools to track the progression of some neurodegenerative diseases and uncovering their pathophysiology [5–8]. By focusing on the study of HSP, our research center established an HSP cohort with clinical, imaging and molecular biomarker records of HSP patients [9]. In this work, we investigated the clinical features, biochemical biomarkers and neuroimaging findings in SPG5. Subsequently, we demonstrate a set of markers, derived and compared sample sizes of each chosen marker. Potentially, one of the most treatable forms of HSP is the SPG5 subtype. But it has received less attention because it is a rare disease. The FDA has provided guidance regarding biomarker use for drug development, especially in rare diseases. Therefore, the main purpose of this work was to estimate the significant parameter(s) that would require the lowest sample size, which has important implications for the design of future therapeutic trials of SPG5.

## Results

### Clinical characteristics of SPG5 patients

Seventeen SPG5 patients, including five with consanguinity, were consecutively enrolled from 14 families. Sixteen patients from 13 unrelated families carried the known nonsense homozygous mutation c.334 C > T (p.R112*). Only one patient carried compound heterozygous mutations c.259 + 2T > C and c.1190C > T (Table 1).

**Table 1:** Clinical and genetic features of SPG5 patients

No	Family	Gender	Mutation cDNA	Consanguineous family/family history	Age at onset(y)	Age at enrollment(y)	Disease duration (y)	SPRS†	Disability score‡
1	F1-II-2	Female	c.259 + 2T > C c.1190C > T	No/No	3	27	24	38	6
2	F2-II-1	Male	c. 334C > T hom	No/No	12	21	9	15	3
3	F3-II-1	Female	c. 334C > T hom	No/No	27	41	14	14	3
4	F4-II-2	Male	c. 334C > T hom	No/No	10	30	20	21	5
5	F5-II-1	Female	c. 334C > T hom	No/No	17	31	14	24	5
6	F6-IV-1	Male	c. 334C > T hom	Yes/No	12	26	14	13	3
7	F7-II-3	Male	c. 334C > T hom	No/No	4	14	10	25	5
8	F8-II-1	Male	c. 334C > T hom	No/No	1	17	16	15	3
9	F9-IV-2	Male	c. 334C > T hom	Yes/Yes	11	34	23	4	2
10	F10-II-3	Male	c. 334C > T hom	No/No	26	42	16	19	4
11	F11-IV-3	Male	c. 334C > T hom	Yes/Yes	8	45	37	26	4
12	F14-II-1	Male	c. 334C > T hom	No/No	11	17	6	11	3
13	F17-II-1	Female	c. 334C > T hom	No/No	10	18	8	14	3
14	F9-IV-1	Female	c. 334C > T hom	Yes/Yes	25	37	12	2	2
15	F11-IV-1	Male	c. 334C > T hom	Yes/Yes	9	49	40	22	5
16	F18-III-1	Male	c. 334C > T hom	No/Yes	5	13	8	8	3
17	F18-II-7	Female	c. 334C > T hom	No/Yes	18	38	20	6	3

No	Harding classification	MMSE	Dorsal column dysfunction	Urinary symptoms	Cerebellar ataxia	Other symptoms			
1	Pure	24	Yes	No	No	No			
2	Pure	27	Yes	No	No	No			
3	Pure	30	Yes	No	No	No			
4	Pure	29	Yes	No	No	No			
5	Pure	29	Yes	No	No	No			
6	Complex	29	Yes	No	No	Epilepsy			
7	Pure	29	Yes	No	No	No			
8	Pure	29	Yes	No	No	No			
9	Pure	29	Yes	No	No	No			
10	Pure	28	Yes	Yes	No	No			
11	Pure	29	Yes	No	No	No			
12	Pure	29	Yes	No	No	No			
13	Pure	29	Yes	No	No	No			
14	Pure	28	Yes	No	No	No			
15	Pure	29	Yes	No	No	No			
16	Pure	27	Yes	No	No	No			
17	Pure	28	Yes	No	No	No			

SPG5, spastic paraplegias type 5; MMSE, mini-mental state examination

^†^Spastic Paraplegia Rating Scale (SPRS), range from zero (no disease manifestation) to a maximum of 52 points (most severe disease manifestation)

^‡^Disability score (0 = no functional handicap, 1 = no functional handicap but signs at examination; 2 = mild, able to run, walking unlimited; 3 = moderate, unable to run, limited walking without aid; 4 = severe, walking with one stick; 5 = walking with two sticks; 6 = unable to walk, requiring wheelchair; 7 = confined to bed)

Age at onset ranged from 1 to 27 years with a median of 11 years. After a median disease duration of 14 years (range 6–40), all patients exhibited a moderate spastic paraplegia with a median Spastic Paraplegia Rating Scale (SPRS) score of 15 (range 2–38). Scores range from zero (no disease manifestation) to a maximum of 52 points (most severe disease manifestation) [10]. According to Harding criteria, based on the clinical phenotype, 16 patients were classified as pure HSP and one patient had complicated HSP with epilepsy. Sixteen patients had severely reduced or nonexistent vibration sense in the lower limbs, and one patient had urinary urgency. None had cognition injury, cerebellar ataxia or axonal peripheral neuropathy (Table 1).

Healthy controls matched SPG5 patients in terms of age [median (range), 30 (13–49) vs 30 (14–50), *P* = 1.000] and gender (male %, 65% vs 65%, *P* = 1.000).

### Spinal cord atrophy measures

**Fig. 1:**
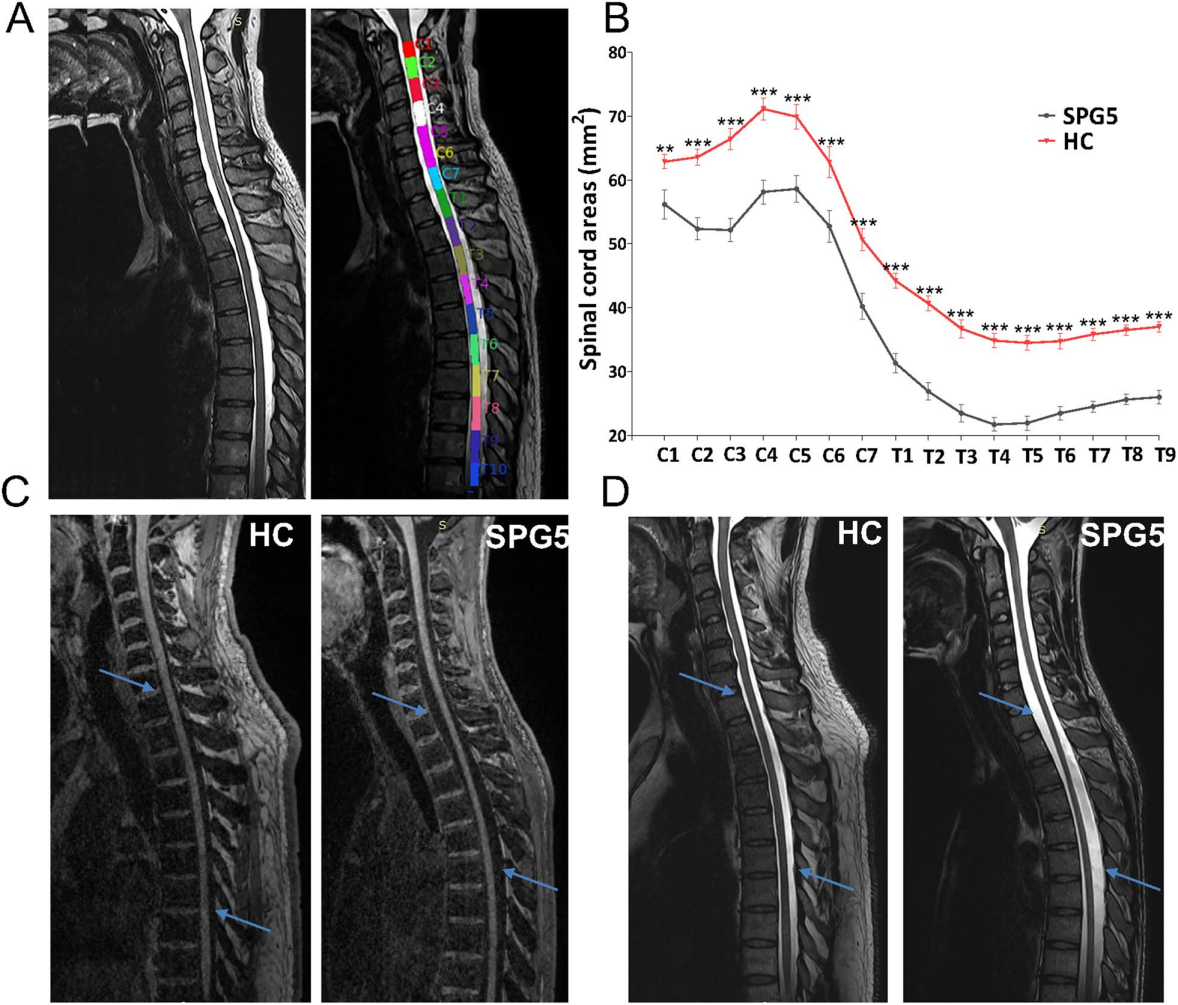
Quantitative MRI to assess spinal cord area. **A** Processing included 3 steps: (1) composing the cervical and thoracic spinal cord with 3D-T2W-SPACE; (2) segmentation and labeling the spinal cord; (3) measurement of spinal cord cross-sectional area (SCA) and sectional diameter (RL and AP). **B** Area of spinal cords (C2-T9) in SPG5 patients and matched healthy controls, comparisons performed using Mann–Whitney test, **P < 0.01, ***P < 0.001. **C**, **D** Representative MRIs of SPG5 and healthy control. In SPG5 patients, a significant thinning in the spinal cord was observed (blue arrow) in the 3D T1-weighted and 3D T2-weighted sequence, respectively

Since the proposed processing pipeline was fully automated, automatic registration into the Spinal Cord Toolbox template was successful in most cases (Fig. 1A). A manual correction was needed for the spinal cord segmentation of only 2 subjects (1 patient and 1 healthy controls).

Spinal cord areas were significantly smaller in SPG5 patients than in controls at all the cervical and thoracic levels evaluated (Fig. 1B–D). Moreover, the relative reduction was more pronounced at thoracic levels (cervical levels: 12–27%; thoracic levels 41–60%), especially in T4 (35 vs 22 mm^2^, *P* < 0.001). Furthermore, we performed ROC analysis to get a diagnostic parameter of spinal cord area at T4. An optimal cut-off value of 25.6 mm^2^ (sensitivity, 88.2%; specificity, 100%) was obtained to differentiate patients with SPG5 from healthy controls, with an AUC of 0.976.

### Conventional MRI findings in the brain

**Fig. 2:**
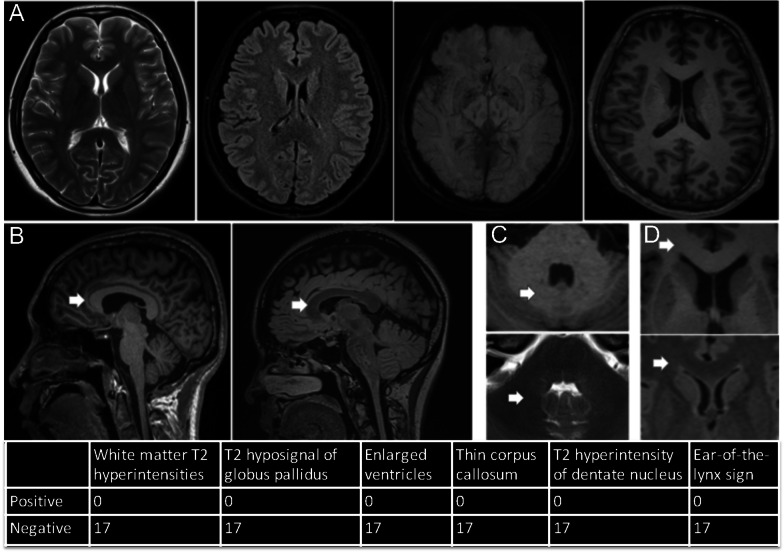
Conventional brain MRI evaluation. **A** Abnormal signals were assessed on T2, FLAIR, SWI and T1 routine sequence (above panel, from left to right). **B**–**D** Neuroradiological signs, previously reported in other subtypes of hereditary spastic paraplegias, were also assessed; **B** thin corpus callosum (left T1, right T2 FLAIR, green edge arrow); **C** T2 hyperintensity of the dentate nucleus (above T1, below T2; black edge arrow); **D** “Ears- of -the- Lynx” MRI sign (above T1, below T2 FLAIR, red edge arrow)

No signal abnormalities were observed on brain T2, fluid attenuated inversion recovery (FLAIR) and susceptibility weighted imaging (SWI) sequences of any SPG5 patients or in healthy controls. “Ear-of-the-lynx” sign, enlarged ventricles, brain white matter T2 hyperintensities, bilateral T2 hypo-signal of the globus pallidus, all previously reported for subtypes of HSP, did not appear in any of these SPG5 patients (Fig. 2).

### Brain atrophy measures

**Fig. 3:**
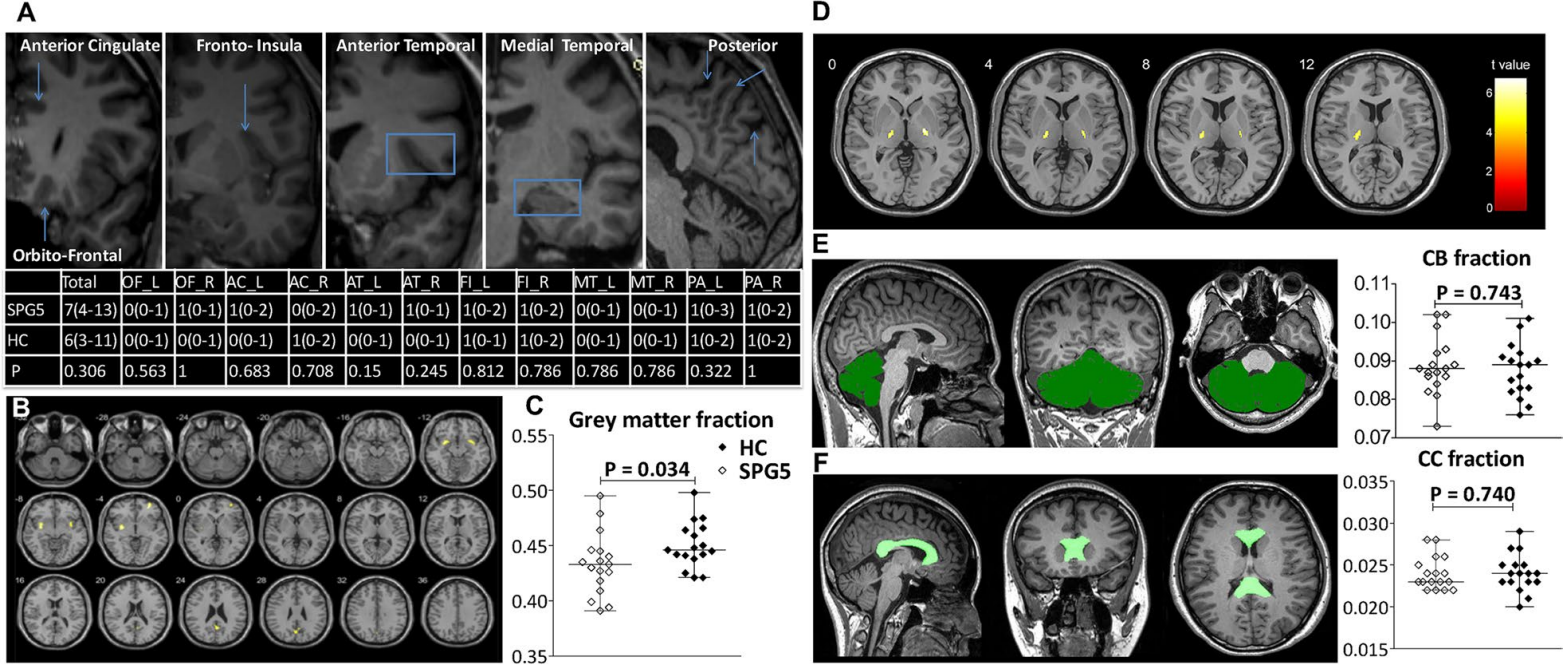
Quantitative MRI to assess the structural changes of brain. **A** Visual rating scales to assess cerebral atrophy, OF = orbitofrontal cortex, AC = anterior cingulate, AT = anterior temporal, FI = fronto-insula, MT = medial temporal lobe, PA = posterior cortex; Visual rating was performed by two neurologists, the intraclass correlation coefficient value ranged from 0.829 to 1.000. Comparisons to each visual rating score and total visual rating scores between the SPG5 patients and the matched healthy controls (HC) were performed using the Mann–Whitney test. **B** Voxel-based morphometry (VBM) was performed to assess brain volume analysis alteration between healthy controls (HC) and SPG5 patients; statistical tests were evaluated at a significance level of *P* < 0.05 corrected for multiple comparisons with the false discovery rate (FDR). **C** Comparisons of the grey matter fraction (GMF) between SPG5 patients and healthy controls were performed using the Mann–Whitney test (right). **D** Voxel-based morphometry (VBM) was performed to assess gray matter analysis alteration between healthy controls (HC) and patients; statistical tests were evaluated at a significance level of *P* < 0.05 corrected for multiple comparisons with FDR. **E**, **F** Comparisons of the cerebellum volume fraction and corpus collosum volume fraction between SPG5 patients and healthy controls were performed using the Mann–Whitney test

Based on MRI visual rating scales, there were no differences in cerebral atrophy assessed with scores for each region or total scores from SPG5 patients compared to healthy controls (Fig. 3A).

Based on voxel measure (Fig. 3B–F), there were no differences in total intracranial volume (TIV, 1517 vs 1564 mL, *P* = 0.652), grey matter volume (GMV, 666 vs 699 mL, P = 0.179), white matter volume (WMV, 539 vs 536 mL, *P* = 0.926) or CSF volume (308 vs 287 mL, *P* = 0.152) compared to healthy controls. Grey matter fraction (GMF) was calculated as, GMF = GMV/TIV, where TIV = GMV + WMV + CSF volume, and a lower GMF in SPG5 patients was observed (0.43 vs 0.45, *P* = 0.03). The GMV was then applied to normalize the raw volumes of the brain structures. The normalized GMV of bilateral thalamus in SPG5 differed from those of healthy controls (peak t value = 6.77, cluster size > 100, *P* < 0.05, false discovery rate (FDR) corrected at cluster-level). No differences were found for cerebellum volume [135 (95–161) vs 132 (114–163) mL, *P* = 0.563] or corpus callosum volume [37 (34–38) vs 37 (35–38) mL, *P* = 0.779), or their volume fractions.

### CSF 27-OHC and NFL measures

The median concentration of CSF 27-OHC was 10 ng/mL (range 7–13) and the median concentration of CSF NFL was 531 pg/mL (range 290–764) in the SPG5 patients. Spearman coefficients demonstrated a positive correlation between CSF 27-OHC and CSF NFL (r = 0.468, *P* = 0.049). There were no correlations between CSF 27-OHC or CSF NFL with the spinal cord area (at any level examined) or any clinical parameters (disease duration, disability, SPRS and MMSE) (*P* > 0.05).

### Sample size estimation

The comparisons and the standardized effect sizes between patients with disease duration > 14 years vs. ≤ 14 years were shown in Additional file 1: Table S1. The selection criterion |t|> 0.5 identified 10 markers, in descending order of |t|: imaging markers including SCA of T9, RL of T9, AP of T9, AP/RL ration of C2, SCA of C1, AP/RL ration of T3, RL of C2, RL of C1, CSF biomarkers including CSF 27-OHC, and clinical assessment of the disability score. Table 2 showed estimated sample size for each marker. The minimum sample size was estimated with the SCA of T9 (n = 22) while the sample size was almost 4 times using CSF 27-OHC (n = 86) and 6 times with disability score (n = 124).

**Table 2:** Sample size estimation

	Effect size	Standard deviation of effect size	Standard effect size |t|	Sample size
SCA-T9	5.08 mm^2^	1.98 mm^2^	1.29	22
RL-T9	0.83 mm	0.32 mm	1.08	30
AP-T9	0.46 mm	0.23 mm	0.99	36
AP/RL-C2	− 0.06	0.04	0.69	68
CSF 27-OHC	− 1.07 ng/ml	0.92 ng/ml	0.62	86
SCA-C1	4.25 mm^2^	4.93 mm^2^	0.59	92
AP/RL-T3	0.03	0.04	0.57	100
RL-C2	0.59 mm	0.43 mm	0.56	104
RL-C1	0.53 mm	0.46 mm	0.52	118
Disability score	− 0.67	0.61	0.51	124

SCA, spinal cord area; RL, right to left diameter of spinal cord; AP, anterior to posterior diameter of spinal cord; AP/RL, AP/RL ratio; CSF, cerebrospinal fluid; 27-OHC, 27-hydroxycholesterol

## Discussion

Comprehensive clinical imaging features and CSF biomarkers of SPG5 have been rarely described previously. Therefore, we undertook the present study to provide a detailed characterization of the structural and CSF signature of SPG5, and to investigate robust markers and derive sample size estimates for HSP clinical trials. To accomplish these goals, we enrolled 17 patients who underwent systematic clinical assessment combined with multimodal MRI evaluation and CSF biomarker evaluation. As expected, total spinal cord area was significantly decreased in compared to healthy controls, regardless of measurement site.

Spinal cord atrophy has been described in a few studies of SPG5 patients or other HSP subtypes [11–13]. However, only a small selection of spinal cross-sectional area or diameter were measured [11–13]. To our knowledge, our study is the first one to thoroughly investigate the whole length of cervical and thoracic regions, with measurements of both cross-sectional area and antero-posterior/ transverse diameter. Our results showed a smaller area of all spinal regions evaluated compared to healthy controls. Further analysis showed that spinal atrophy was more pronounced in the thoracic than the cervical spinal cord, consistent with the pattern of spinal atrophy in autosomal dominant HSP. This may partially explain the common clinical manifestation of symptomatic lower limb spasticity without upper limb involvement [12].

No abnormalities were found by conventional brain MRI or quantitative brain MRI-derived measures, except for a mildly reduced gray matter on the thalamus. In general, specific MRI characteristics of the brain are helpful to differentiate SPG types [2]. White matter lesions without unified patterns were commonly seen in SPG5 patients in previous studies [11, 14–16], and mild cerebellar atrophy was reported as well [11]. Surprisingly, we failed to identify any cerebral signal abnormalities on conventional MRI or any clear cerebral atrophy through two advanced imaging techniques (visual rating of cerebral atrophy and quantitative brain measures). These discrepancies might be partially explained by the specific genotype of the patients enrolled (most were homozygous c.334 C > T [p.R112*] mutation), the more patients with pure form, a relatively shorter disease duration and different imaging techniques used in our study. Nonetheless, further studies are warranted to explore the brain imaging characteristics in SPG5 patients.

In addition, we explored the spinal cord atrophy and disease duration or severity of SPG5 from a molecular-pathogenesis perspective. The accumulation of 27-OHC may not only be a biomarker but also a key factor driving tissue damage in patients with SPG5 [3]. NFL is a protein component of the cytoskeleton of myelinated axons, and as such, constitutes a putative biomarker to reflect axonal injury [8, 17]. Furthermore, the concentration of CSF 27-OHC in SPG5 in this study was higher than previously reported levels of healthy controls (CSF 27-OHC, 0.5 ng/mL^3^) [3], and was positively correlated with the CSF NFL concentration. These results could indicate that neurotoxic 27-OHC is associated with axonal injury. However, there was no correlation between the concentrations of 27-OHC or NFL in the CSF with the progressive degeneration reflected by worsening imaging indicators (spinal cord area) or disease duration or severity (clinical parameters). Therefore, we proposed that although CSF 27-OHC and CSF NFL may be suitable markers for monitoring disease activities, further well-designed studies with large sample size are warranted to explore whether they are useful guides to the overall progression of SPG5 like multiple sclerosis [18, 19].

Furthermore, we set the median disease duration 14 years as a cut-off value to monitor disease progression and identified a set of markers that showed standardized effect size |t|> 0.5. The current sample size estimates were based on the assumption that a given treatment could result in slower disease progression for 14 years. The results suggested that SCA measurements of T9 required the lowest number of participants, followed by CSF biomarkers, and the clinical disability assessment requiring highest sample size. Thus, we propose SCA of T9 as potential candidate for clinical trial endpoints. Such estimations are crucial for therapeutic testing against rare disease such as SPG5, where patient recruit is difficult and large sample sizes unpractical.

The use of detailed spinal cord morphometry provides a whole picture of spinal cord atrophy at each level in SPG5 patients. The combination of imaging characteristics with CSF biomarkers is helpful to explore the underlying mechanisms driving disease progression. Nonetheless, this study presents several limitations as well. From a statistical standpoint, the relatively small sample size and cross-sectional study design, forced us to set the median disease duration as the cut-off value for the assumed clinical trial. However, such treatment duration of 14 years would quite long in real clinical practice. The absolute reduction of SCA on T9 segment was approximately 5 mm^2^ with a standard deviation of 2 mm^2^. Therefore, more sensitive biomarkers are needed in future exploratory interventional studies to monitor SPG5 progression. It remains uncertain whether this approach could reliably reflect disease progression, which should be tested in future longitudinal study. Due to the rarity of HSP, further cooperation between different centers is needed. Finally, imaging modalities as those here presented are relatively conventional and more advanced imaging techniques like diffusion tensor imaging may provide more detailed evidence of pathogenesis.

## Conclusions

In conclusion, spinal cord atrophy was more severe in the thoracic level than cervical level in the SPG5 patients, no apparent cerebral abnormalities are observed in our patients, and our findings support measurement of SCA on T9 as a primary endpoint clinical trials with a relatively long duration. Efforts should be focused to explore new sensitive and specific imaging features or biomarkers to track disease changes in future studies.

## Methods

### Subjects

We used a cross-sectional design for comparing SPG5 patients to age- and sex-matched healthy controls. The SPG5 patients stemmed from an HSP cohort at the Neurogenetic Diseases Centers in the First Affiliated Hospital of Fujian Medical University at Fuzhou, China. Patients with clinically manifested HSP and genetically confirmed diagnosis of SPG5 were eligible to participate in this study. Exclusion criteria were: patients with (1) other neurologic or systemic diseases, (2) substance abusers, (3) other causes of focal or diffuse brain and spinal cord damage at routine MRI sessions (4) restrictions for MRI scanning and (5) restrictions for lumbar puncture. Of the 34 patients considered for this trial, 17 met all criteria specified.

Healthy controls underwent neurologic evaluation and MRI assessment and were included only if they had normal findings.

### Clinical and biochemical assessments

Clinical data, CSF and blood were collected for analysis according to standardized protocols. On the day of MRI scanning, each patient had a full neurological evaluation by an experienced observer, including: SPRS, to quantify severity of disease [10]. disability score, to assess deformity level [20]; Harding classification, to classify pure or complex pattern [21]; and mini-mental state examination (MMSE) to detect cognitive injury. After overnight fasting (12 h), patients underwent blood collection by venipuncture at room temperature. Simultaneously, CSF was also collected via lumbar puncture, and all samples were stored at − 80 °C until use. CSF 27-OHC was analyzed by using ultra-performance liquid chromatography-tandem mass spectrometry (UPLC-MS/MS) and quantified with a stable isotope dilution method. CSF NFL was measured using the Simoa NF-Light Advantage Kit (Quanterix) on a Simoa HD-1 Analyzer instrument, according to the manufacturer’s instructions.

### MRI protocols

All subjects were examined with a 3T Siemens scanner (MAGNETOM Skyra) equipped with a 20-channel head-neck coil and a 24-channel spine-array coil. Cervical and thoracic spinal cord images were obtained by using 3D T2-weighted turbo spin-echo sequence (Sampling Perfection with Application-optimized Contrast by using different flip angle Evolutions, SPACE) and 3D-T1-weighted turbo spin-echo sequence (magnetization prepared rapid acquisition gradient echo, MPRAGE) in the sagittal plane. Brain images were obtained with 3D T1- MPRAGE in the sagittal plane and T2, FLAIR and SWI in the axial plane. The parameters of main MRI sequences are listed in Additional file 1: Table S2.

### Image analyses

#### Spinal cord cross-sectional area measurement

After completion of the MRI scans, the 3D T2 images of cervical and thoracic spine areas were simultaneously stitched to ensure full presentation of each cervicothoracic spinal cord (C1-T9) acquired by using Compose software on a SIEMENS workstation (Fig. 1A). Spinal cord segmentation and cross-sectional area (SCA) measurements were performed by using the Spinal Cord Toolbox, Version 4.01 (https:// sourceforge.net/projects/spinalcordtoolbox/). Morphological metrics of the spinal cord at each vertebral level (from C1 to T9), including SCA and sectional diameter (anterior–posterior AP, right-left RL, AP/RL ratio), were extracted for further analysis.

#### Conventional brain MRI evaluation

Abnormal signals were assessed on T2, T1, FLAIR and SWI routine sequences. Some neuroradiological signs, which have been previously reported in specific subtypes of HSP [11], were also analyzed in the current SPG5 patients.

#### Visual rating of cerebral atrophy

Visual rating of 3D T1 images from each participant was performed by two independent neurologists, trained in the consistency of scales evaluation. Images were rated in native space with the RadiAnt DICOM Viewer. Six regions were rated based on existing scales and a simplified version with a detailed evaluation protocol, as previously described [22].

#### Quantitative brain MRI evaluation

Voxel-based morphometry (VBM) was performed to assess brain volume and GMV alteration of patients compared to healthy controls. The anatomical automatic labeling (AAL) brain atlas template was used to extract the volume of region of interest (ROI). The GMV of each voxel was processed by using the CAT12 toolbox (http://dbm.neuro.uni-jena.de/cat/), incorporated in SPM12 (www.fil.ion.ucl.ac.uk/spm) running under MATLAB R2016a. Corpus callosum volumes were calculated using a script file written by Ged Ridgway (http://www0.cs.ucl.ac.uk/staff/g.ridgway/vbm/get_totals.m), running under MATLAB R2016a. Statistical tests were evaluated at a significance level of *P* < 0.05, corrected for multiple comparisons with the FDR and family-wise error rate (FWE) at cluster level and cluster size, which must be greater than 100 voxels. Cerebellum volume was also measured with MRIconN (https://www.nitrc.org/projects/mricron).

### Statistical analysis

The results are expressed as medians with ranges for continuous variables and probability for categorical variables. Kolmogorov–Smirnov tests were used to assess normality of the variables. Differences were assessed using Student’s *t*-test for normally distributed variables, otherwise using the Mann–Whitney test. Categorical variables were compared for the groups using the *Chi*-squared test (Fisher’s exact test when the expected value is < 5). The associations were assessed with Pearson correlation analysis for normally distributed variables, otherwise using Spearman correlation analysis. Significant group differences at *P < 0.05. P-values are Bonferroni-adjusted for multiple comparisons.

Statistical analysis of structural image data was conducted as follows: voxel-based independent two samples t-test was performed to analyze differences between groups in GMV between healthy controls and SPG5 patients. Statistical analysis was performed with SPSS 25.

The calculation of sample size for a hypothetical clinical trial assumed that a given treatment could result in slower disease progression over a median disease duration period. Due to the small sample size, we divided the patients by median split according to the disease duration in order to make sure that the numbers of patients in the two groups were comparable. Standardized effect sizes were estimated for each clinical variable, CSF biomarkers and quantitative MRI features separately. Standardized effect sizes |t| were calculated as the differences between means of two groups, divided by the residual standard deviation in the group of patients with longer than median disease duration. The |t|> 0.5 threshold was applied to select potential markers following the rule of thumb proposed by Cohen to identify small (0.2), medium (0.5) and large (0.8) effect sizes [23]. An 80% power level was used and two-side 0.05 level was considered significant. Statistical analysis was performed with PASS 15.

## Supplementary Information

**Additional file 1.Table S1.** Comparisons and standardized effect size between SPG5 patients with disease duration > 14 years and ≤ 14 years. **Table S2.** The parameters of MR sequences.

## Data Availability

A fully anonymized version of the dataset used for analysis with individual participant data and a data dictionary will be available for other researchers to apply to use, via https://research.yiducloud.com.cn/#/project/list?desease=research_nervous_standard. Written proposals will be assessed by corresponding author of this study (linyi7811@163.com) and a decision made about the appropriateness of the use of data.

## Abbreviations

SPG5: Hereditary spastic paraplegias type 5
HSP: Hereditary spastic paraplegias
27-OHC: 27-Hydroxycholesterol
NFL: Neurofilament light chain
CSF: Cerebrospinal fluid
MRI: Magnetic resonance imaging
SCA: Spinal cord area
TIV: Total intracranial volume
GMV: Grey matter volume
GMF: Grey matter fraction
SPRS: Spastic Paraplegia Rating Scale
MMSE: Mini-mental state examination
UPLC-MS: Ultra-performance liquid chromatography-tandem mass spectrometry
SPACE: Sampling Perfection with Application-optimized Contrast by using different flip angle Evolutions
MPRAGE: Magnetization prepared rapid acquisition gradient echo
FLAIR: Fluid attenuated inversion recovery
SWI: Susceptibility weighted imaging
AP: Anterior-posterior
RL: Right-left
VBM: Voxel-based morphometry
GMV: Grey matter volume
AAL: Anatomical automatic labeling
ROI: Region of interest
FDR: False discovery rate
FEW: Family-wise error rate
