# Correction to: Potential markers for sample size estimations in hereditary spastic paraplegia type 5

**DOI:** 10.1186/s13023-021-02078-8

**Published:** 2021-10-27

**Authors:** Qianqian Lin, Ying Liu, Zhixian Ye, Jianping Hu, Wenjie Cai, Qiang Weng, Wan-Jin Chen, Ning Wang, Dairong Cao, Yi Lin, Ying Fu

**Affiliations:** 1 Department of Neurology and Institute of Neurology of First Affiliated Hospital, Institute of Neuroscience, Fujian Medical University, Fuzhou, 350005 China; 2 Fujian Key Laboratory of Molecular Neurology, Fujian Medical University, Fuzhou, 350005 China; 3 Department of Radiology of First Affiliated Hospital, Fujian Medical University, Fuzhou, 350005 China

## Correction to: Orphanet J Rare Dis (2021) 16:391 10.1186/s13023-021-02014-w

Following the publication of the original article [1] it was brought to our attention that author Dairong Cao’s affiliation had inadvertently been changed to “2. Fujian Key Laboratory of Molecular Neurology, Fujian Medical University, Fuzhou 350005, China” during the implementation of the author’s corrections.

Author Dairong Cao’s correct affiliation is “3. Department of Radiology of First Affiliated Hospital, Fujian Medical University, Fuzhou 350005, China”.

This is shown next to the author’s name in the author list of this Correction and has also been updated in the original article.
