# Supplementary material for: Potential markers for sample size estimations in hereditary spastic paraplegia type 5
Source: Orphanet J Rare Dis. 2021 Sep 19;16:391. doi: 10.1186/s13023-021-02014-w (PMC8451094; doi:10.1186/s13023-021-02014-w)
Supplement: Supplementary file 1 — Additional file 1.Table S1. Comparisons and standardized effect size between SPG5 patients with disease duration > 14 years and ≤ 14 years. Table S2. The parameters of MR sequences. [file 13023_2021_2014_MOESM1_ESM.docx]

| **Table S1 Comparisons and standardized effect size between SPG5 patients with disease duration > 14 years and ≤14 years** | | | | |
| --- | --- | --- | --- | --- |
| **Variables** | **SPG5 patients with disease duration ≤ 14 years (n=9)** | **SPG5 patients with disease duration > 14 years (n=8)** | ***P* value** | **Standardized effect size \|t\|** |
| **Age at onset(years**±**SD)** | 13.67±8.00 | 10.75±8.03 | 0.465 | 0.36 |
| **Male, n(%)** | 5(55.6) | 6(75.0) | 0.620 | / |
| **Clinical assessment** | | | | |
| SPRS | 14.00±7.18 | 18.88±10.91 | 0.288 | 0.45 |
| Disability | 3.33±1.00 | 4.00±1.31 | 0.253 | 0.51 |
| **Imaging assessment (mean±SD)** | | | | |
| AP-C1 | 7.30±0.96 | 7.16±0.53 | 0.724 | 0.26 |
| AP-C2 | 6.54±0.59 | 6.68±0.66 | 0.673 | 0.20 |
| AP-C3 | 6.17±0.50 | 6.18±0.54 | 0.974 | 0.02 |
| AP-C4 | 6.27±0.48 | 6.26±0.52 | 0.987 | 0.01 |
| AP-C5 | 6.18±0.43 | 6.30±0.59 | 0.635 | 0.21 |
| AP-C6 | 5.84±0.51 | 5.94±0.60 | 0.735 | 0.16 |
| AP-C7 | 5.24±0.48 | 5.29±0.61 | 0.873 | 0.07 |
| AP-T1 | 4.82±0.39 | 4.78±0.55 | 0.839 | 0.839 |
| AP-T2 | 4.61±0.34 | 4.60±0.57 | 0.961 | 0.02 |
| AP-T3 | 4.41±0.43 | 4.40±0.61 | 0.966 | 0.02 |
| AP-T4 | 4.28±0.39 | 4.26±0.64 | 0.953 | 0.02 |
| AP-T5 | 4.36±0.46 | 4.36±0.65 | 0.980 | 0.01 |
| AP-T6 | 4.59±0.42 | 4.51±0.65 | 0.775 | 0.12 |
| AP-T7 | 4.78±0.36 | 4.70±0.51 | 0.717 | 0.15 |
| AP-T8 | 4.88±0.32 | 4.94±0.50 | 0.770 | 0.12 |
| AP-T9 | 5.21±0.41 | 4.75±0.47 | 0.046 | 0.99 |
| RL-C1 | 10.16±0.70 | 9.63±1.02 | 0.225 | 0.52 |
| RL-C2 | 10.39±0.53 | 9.80±1.05 | 0.158 | 0.56 |
| RL-C3 | 11.00±0.55 | 10.58±1.34 | 0.425 | 0.32 |
| RL-C4 | 12.04±0.49 | 11.78±1.52 | 0.645 | 0.18 |
| RL-C5 | 12.01±0.84 | 12.03±1.36 | 0.980 | 0.01 |
| RL-C6 | 11.31±1.22 | 11.49±1.47 | 0.791 | 0.12 |
| RL-C7 | 9.62±1.10 | 9.80±1.31 | 0.765 | 0.14 |
| RL-T1 | 8.26±0.76 | 8.38±1.13 | 0.799 | 0.11 |
| RL-T2 | 7.41±0.90 | 7.45±0.94 | 0.932 | 0.04 |
| RL-T3 | 6.69±1.20 | 6.84±0.87 | 0.776 | 0.17 |
| RL-T4 | 6.64±0.41 | 6.41±0.67 | 0.395 | 0.35 |
| RL-T5 | 6.54±0.49 | 6.33±0.71 | 0.466 | 0.31 |
| RL-T6 | 6.67±0.57 | 6.45±0.73 | 0.505 | 0.29 |
| RL-T7 | 6.69±0.33 | 6.58±0.68 | 0.675 | 0.17 |
| RL-T8 | 6.79±0.35 | 6.54±0.57 | 0.285 | 0.44 |
| RL-T9 | 6.99±0.40 | 6.16±0.76 | 0.012 | 1.08 |
| AP/RL-C1 | 0.72±0.07 | 0.75±0.09 | 0.445 | 0.35 |
| AP/RL-C2 | 0.63±0.05 | 0.69±0.08 | 0.099 | 0.69 |
| AP/RL-C3 | 0.56±0.04 | 0.59±0.08 | 0.298 | 0.41 |
| AP/RL-C4 | 0.52±0.04 | 0.54±0.08 | 0.620 | 0.20 |
| AP/RL-C5 | 0.52±0.04 | 0.53±0.06 | 0.599 | 0.22 |
| AP/RL-C6 | 0.52±0.04 | 0.52±0.06 | 0.920 | 0.05 |
| AP/RL-C7 | 0.55±0.04 | 0.55±0.06 | 0.852 | 0.08 |
| AP/RL-T1 | 0.58±0.05 | 0.57±0.05 | 0.660 | 0.20 |
| AP/RL-T2 | 0.63±0.07 | 0.62±0.04 | 0.841 | 0.14 |
| AP/RL-T3 | 0.67±0.09 | 0.64±0.05 | 0.411 | 0.57 |
| AP/RL-T4 | 0.65±0.05 | 0.67±0.06 | 0.547 | 0.28 |
| AP/RL-T5 | 0.67±0.06 | 0.69±0.05 | 0.579 | 0.30 |
| AP/RL-T6 | 0.69±0.06 | 0.70±0.06 | 0.825 | 0.11 |
| AP/RL-T7 | 0.72±0.05 | 0.72±0.07 | 0.947 | 0.03 |
| AP/RL-T8 | 0.72±0.05 | 0.754±0.07 | 0.304 | 0.47 |
| AP/RL-T9 | 0.75±0.05 | 0.77±0.06 | 0.380 | 0.39 |
| SCA-C1 | 58.18±10.89 | 53.93±7.15 | 0.363 | 0.59 |
| SCA-C2 | 53.32±6.59 | 51.23±7.93 | 0.560 | 0.26 |
| SCA-C3 | 53.16±6.00 | 51.01±9.06 | 0.569 | 0.24 |
| SCA-C4 | 58.87±5.75 | 57.25±9.91 | 0.682 | 0.16 |
| SCA-C5 | 58.10±7.07 | 59.11±10.59 | 0.818 | 0.10 |
| SCA-C6 | 51.88±9.43 | 47.70±22.33 | 0.615 | 0.19 |
| SCA-C7 | 39.82±7.89 | 40.70±9.24 | 0.835 | 0.09 |
| SCA-T1 | 31.21±4.94 | 31.50±7.59 | 0.926 | 0.04 |
| SCA-T2 | 26.77±4.71 | 27.09±6.84 | 0.911 | 0.05 |
| SCA-T3 | 23.44±5.32 | 23.63±6.28 | 0.950 | 0.03 |
| SCA-T4 | 22.13±3.15 | 21.34±5.78 | 0.725 | 0.14 |
| SCA-T5 | 22.18±3.51 | 21.75±5.75 | 0.854 | 0.07 |
| SCA-T6 | 24.02±3.69 | 22.96±5.51 | 0.644 | 0.19 |
| SCA-T7 | 24.90±2.64 | 24.18±4.46 | 0.685 | 0.16 |
| SCA-T8 | 25.98±2.61 | 25.29±4.17 | 0.684 | 0.17 |
| SCA-T9 | 28.44±3.54 | 23.36±3.95 | 0.014 | 1.29 |
| **CSF biomarkers** | | | | |
| NFL | 529.22±137.09 | 549.50±82.43 | 0.721 | 0.25 |
| 27-OHC | 9.02±1.76 | 10.09±1.72 | 0.228 | 0.62 |
| Significant group differences at * *P*< 0.05. Bonferroni-adjusted correction for imaging assessment * *P*< 0.0008.  Abbreviations: SPG5, hereditary spastic paraplegia type 5; SPRS, Spastic Paraplegia Rating Scale; AP, anterior to posterior diameter of spinal cord; RL, right to left diameter of spinal cord; AP/RL, AP/RL ratio; SCA, spinal cord area; CSF, cerebrospinal fluid; 27-OHC, 27-hydroxycholesterol; NFL, neurofilament; 27-OHC, 27-hydroxycholesterol. | | | | |

| **Table S2 The parameters of MR sequences** | | | | | | | |
| --- | --- | --- | --- | --- | --- | --- | --- |
| sequence | TR(ms) | TE(ms) | FOV(mm) | Voxel Size (mm) | Flip angle (deg) | Average | TA min:sec |
| **Spinal cord** | | | | | | | |
| 3D -T1-MPRAGE | 2300 | 3.43 | 250×250 | 1.0×1.0×0.8 | 9 | 1 | 5:21 |
| 3D-T2-SPACE | 1500 | 135 | 250×250 | 0.4×0.4×0.8 | 140 | 1.4 | 5:54 |
| **Brain** | | | | | | | |
| 3D -T1-MPRAGE | 2300 | 2.32 | 240×240 | 0.9×0.9×0.9 | 8 | 1 | 5:21 |
| 2D-T2 | 6000 | 125 | 220×220 | 0.6×0.6×5.0 | 90 | 1 | 1:12 |
| 2D-FLAIR | 8500 | 81 | 220×220 | 0.7×0.7×3.0 | 150 | 1 | 2:16 |
| 2D-SWI | 27 | 20 | 240×240 | 0.9×0.9×2.0 | 150 | 1 | 2:15 |
| Abbreviations: TR, repetition time; TE, echo time; FOV, field of view; TA, average time; 3D-T1-MPRAGE, three-dimensional T1 weighted magnetization prepared rapid gradient echo sequences; 3D-T2-SPACE, three-dimensional T2 weighted Sampling Perfection with Application-optimized Contrast by using different flip angle Evolutions; FLAIR, fluid attenuated inversion recovery; SWI, susceptibility weighted imaging. | | | | | | | |
